# Global trends in *Trichoderma* secondary metabolites in sustainable agricultural bioprotection

**DOI:** 10.3389/fmicb.2025.1595946

**Published:** 2025-07-18

**Authors:** Anthony Apolinario Cortez-Lázaro, Pedro James Vázquez-Medina, Edson Max Caro-Degollar, Jennifer Valeria García Evangelista, Ronald Alexis Cortez-Lázaro, Jorge Luis Rojas-Paz, Jose Antonio Legua-Cardenas, Fredesvindo Fernandez-Herrera, Carlos Roberto Pesantes-Rojas, Robert William Ocrospoma-Dueñas, Segundo Manuel Oliva-Cruz, Gabriel Alberto Manes-Cangana, José Luis Romero Bozzetta, Santos Triunfo Leiva Espinoza

**Affiliations:** ^1^Grupo de investigación en procesos en Sanidad Vegetal, Instituto de Investigación para el Desarrollo Sustentable de Ceja de Selva, Universidad Nacional Toribio Rodríguez de Mendoza, Chachapoyas, Perú; ^2^Facultad de Ciencias, Universidad Nacional José Faustino Sánchez Carrión, Huacho, Perú; ^3^Escuela Profesional de Agronomía, Facultad de Ciencias Agrarias, Universidad Nacional de Cañete, San Vicente de Cañete, Perú; ^4^Escuela de Ingeniería Química, Facultad de Ingeniería Química y Metalurgia, Universidad Nacional José Faustino Sánchez Carrión, Huacho, Perú

**Keywords:** bibliometrics, biological control, secondary metabolites, *Trichoderma* spp., agricultural sustainability, plant health

## Abstract

The use of *Trichoderma* spp. constitutes a promising biotechnological strategy for sustainable agriculture, owing to its capacity to control phytopathogens and to produce bioactive secondary metabolites. This study, one of the first of its kind, addresses the absence of a comprehensive bibliometric assessment in this field. A systematic bibliometric analysis was conducted on 235 publications indexed in Scopus (2000–2025). Advanced tools such as VOSviewer and Bibliometrix were employed to track publication trends, identify key research themes, map collaborative networks, and assess the influence of leading authors and institutions. An exponential increase in scientific output was observed, peaking in 2023. Four principal research clusters were identified: antifungal activity, gene regulation, secondary metabolite production, and biosynthesis. India and China accounted for the highest publication volume, while Italy, represented by authors such as Francesco Vinale, accounted for the greatest scientific impact. International collaboration was extensive, particularly between Asia and Europe. The analysis indicates a progression from applied biocontrol studies to research focusing on molecular and genetic mechanisms, highlighting the need for multidisciplinary approaches that integrate biotechnology, agronomy, and microbial ecology. This bibliometric study provides an overview of *Trichoderma* secondary metabolites in agricultural biocontrol and outlines a research agenda emphasizing field validation, interdisciplinary collaboration, and the adoption of innovative technologies to bridge the gap between research and on-farm application in sustainable agriculture.

## 1 Introduction

Global food demand is rising exponentially, while natural resources are becoming increasingly scarce, creating an urgent need for sustainable agricultural practices to ensure food security and environmental sustainability (Sala et al., 2021; Seenivasagan and Babalola, 2021). This challenge is further exacerbated by the presence of plant pathogens, which cause significant crop losses and threaten food security (Chávez-Díaz et al., 2020). For decades, pathogen control has relied heavily on chemical pesticides, which, despite their initial effectiveness, have proven unsustainable due to their environmental impact and the emergence of resistant pathogen strains (María Suaste, 2020; Ribeiro et al., 2001). In this context, the search for biological alternatives has become a scientific and global priority.

Among these alternatives, *Trichoderma*, a widely studied fungal genus, has emerged as a promising tool for agricultural bioprotection (Bailey et al., 2008; Fontana et al., 2021; Vinale et al., 2006). *Trichoderma* is distinguished by its dual capacity to compete with pathogens and produce a diverse array of bioactive secondary metabolites that play critical roles in biocontrol and plant health (Buojaylah et al., 2024; Rajesh et al., 2016; Savas et al., 2021; Manzar et al., 2022). These metabolites, such as peptaibols, terpenoids and pyrones, not only act as antifungals and antibacterials, but also modulate the soil microbiota, promoting an ecological balance that benefits both plants and the agricultural ecosystem (Hermosa et al., 2014; Khan et al., 2020; Reino et al., 2008).

The particularity of these metabolites lies in their dual function: on the one hand, they act directly against phytopathogens, altering the permeability of their cell membranes or inhibiting their growth (Alfaro-Vargas et al., 2022; Shi et al., 2012; Zhang et al., 2022); on the other hand, they activate plant defense mechanisms, such as Systemic Acquired Resistance (SAR) and Induced Systemic Resistance (ISR), enhancing the plant’s ability to resist infections and abiotic stresses (Sharma et al., 2019; Shoresh et al., 2010; Visagie et al., 2023). This duality makes *Trichoderma* a promising alternative for sustainable agriculture.

However, despite its potential, the application of these metabolites under field conditions still faces significant challenges. Factors such as genetic variability among strains, environmental conditions, and the incomplete understanding of the regulatory mechanisms involved limit their efficacy and stability (López-Bucio et al., 2015; Poveda et al., 2020; Zeilinger et al., 2016).

In addition, although several studies have investigated *Trichoderma* metabolites and their biocontrol capacity, a comprehensive bibliometric analysis has yet to be conducted to provide a detailed mapping of the evolution of research in this field. This information gap prevents a clear understanding of major scientific trends, the development of innovative methodologies, and the identification of critical gaps in the literature (Hood and Wilson, 2001). Bibliometrics, as a powerful tool for analyzing large volumes of scientific data, offers an opportunity to comprehend the evolution of research in this domain and its prospective trajectory (Aria and Cuccurullo, 2017; van Eck and Waltman, 2010).

This study aims to address this gap by conducting a comprehensive bibliometric analysis of the scientific literature on *Trichoderma* secondary metabolites in agricultural bioprotection, using data indexed in Scopus. For this purpose, advanced tools such as VOSviewer and Bibliometrix will be used to identify the main lines of research, determine the impact of the most influential scientific contributions, and detect emerging areas as well as critical gaps to guide future research.

## 2 Materials and methods

### 2.1 Search strategy and data selection

**Figure 1:**
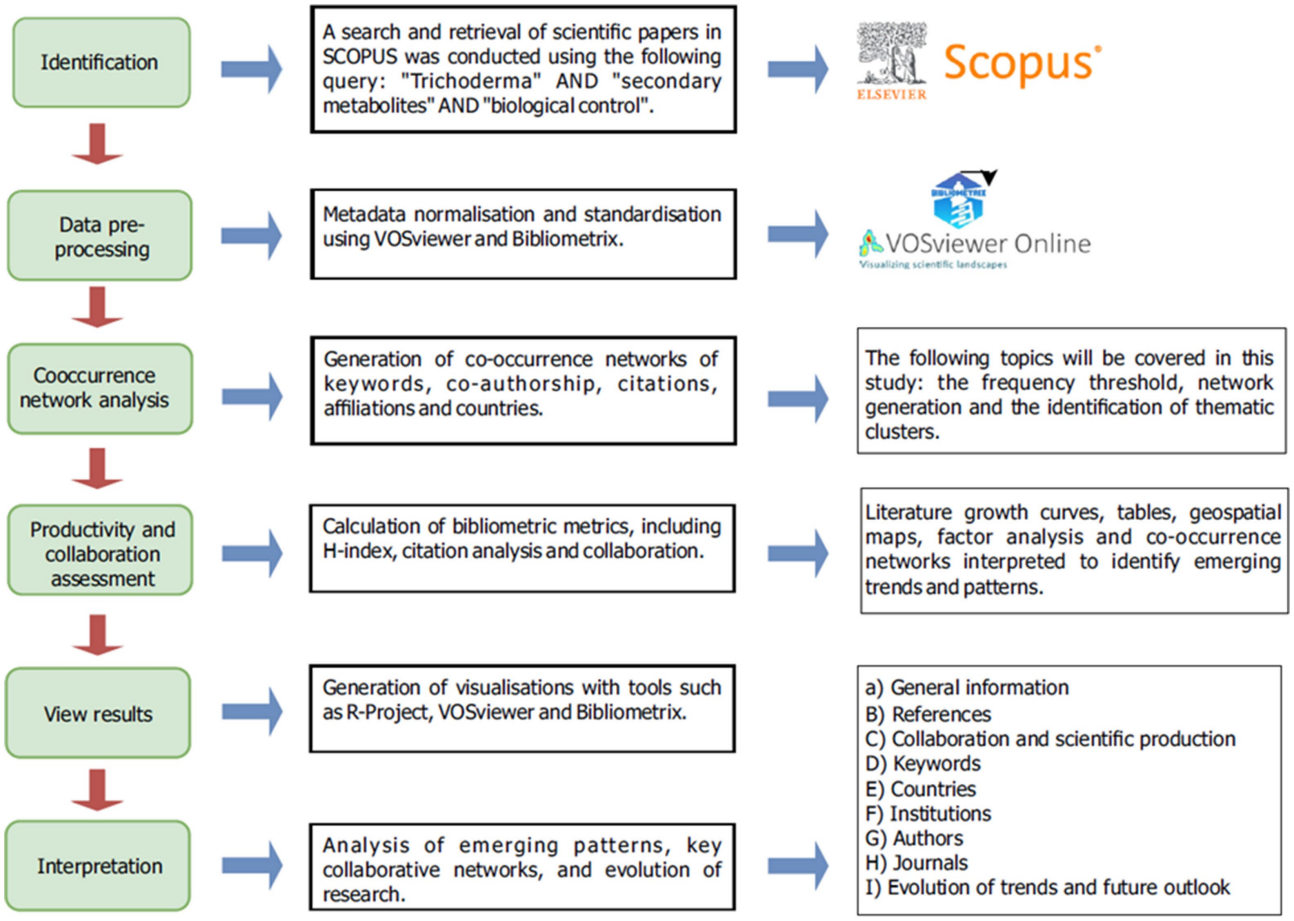
Flow chart of bibliometric analysis procedures, information retrieval strategy, analysis tools and main indicators. Source: Own elaboration.

The bibliometric analysis was conducted using the Scopus database (Elsevier), selected for its comprehensive coverage of agricultural microbiology and biotechnology literature, as well as for its capacity to provide detailed metadata and compatibility with advanced network analysis and citation tools (Figure 1). The search was conducted on January 24, 2025, covering all publications indexed up to that date. To ensure an exhaustive retrieval of scientific papers, the following query was used in the Title, Abstract, and Keywords (TITLE-ABS-KEY) fields:

*Trichoderma* AND secondary metabolites AND biological control.

No restrictions were applied regarding language, document type, or subject category to ensure the inclusion of all relevant literature available in Scopus. The retrieved records were exported in CSV format, including metadata such as titles, abstracts, keywords, author affiliations, cited references, and citation metrics.

### 2.2 Data processing and analysis

The data obtained were processed using the tools VOSviewer (v.1.6.19) (van Eck and Waltman, 2010) and Bibliometrix (Aria and Cuccurullo, 2017) selected for their efficiency in handling large volumes of data and their ability to generate complex network visualizations. In VOSviewer, keyword co-occurrence networks were generated, with a minimum frequency threshold established depending on the type of analysis, allowing for the identification of thematic clusters and emerging trends. Co-authorship and institutional collaboration networks were constructed using the association method available in VOSviewer, with the purpose of detecting relevant scientific communities in the field of study. In addition, productivity and impact metrics were calculated, including the H-index of authors and institutions, the geographical distribution of publications, and the temporal evolution of scientific production.

### 2.3 Visualization and synthesis

The results were synthesized into visual representations to facilitate the interpretation of research dynamics in the study area. Growth curves of the literature were generated using the R-Project software (version 4.4.3), applying a second-degree polynomial model to evaluate the increase in the number of studies and the emergence of new lines of research. Networks of co-occurring terms were analyzed to identify emerging patterns and predominant areas of research. Co-citation analysis was performed to identify the most influential articles and authors in research on *Trichoderma* secondary metabolites and their role in biocontrol. To evaluate the geographical contribution to scientific production, geospatial maps were constructed in Bibliometrix using affiliation data provided by Scopus, which allowed for the identification of the global distribution of publications and collaboration between countries, as described in Figure 1.

## 3 Results

### 3.1 Scopus bibliometric indicators (2000–2025)

**Figure 2:**
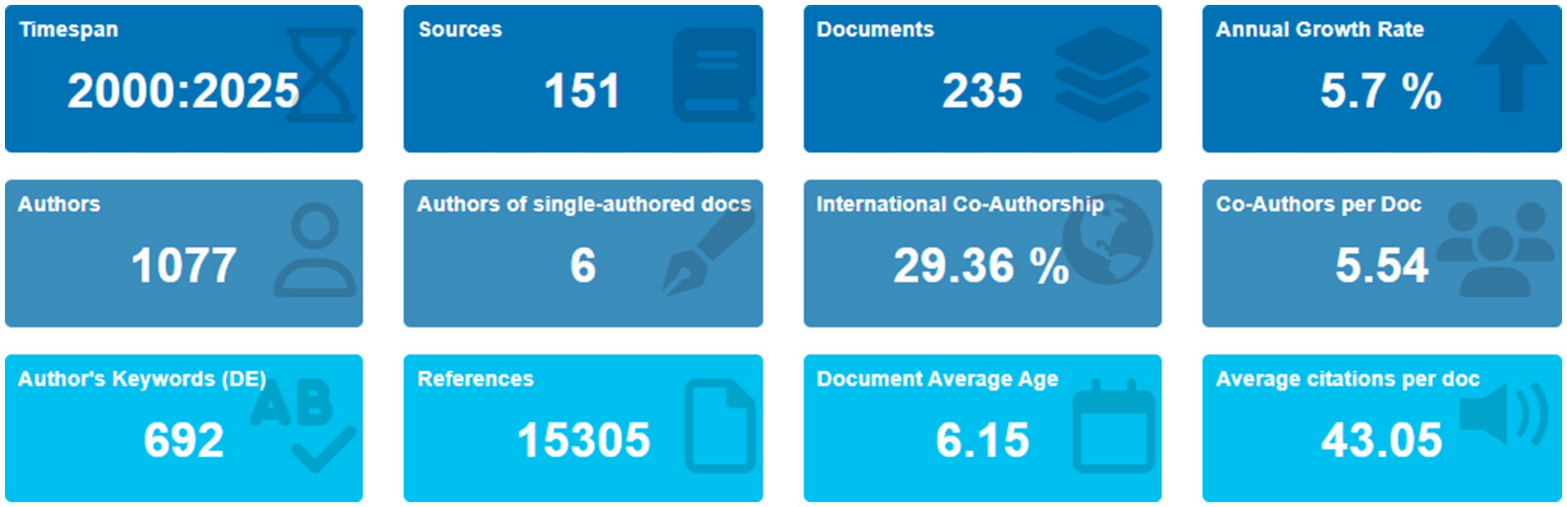
Summary of bibliometric indicators on *Trichoderma* and its metabolites in biological control (2000–2025). Source: Own elaboration based on data from SCOPUS using Bibliometrix software.

Indicators of the literature indexed in Scopus on *Trichoderma* and its secondary metabolites in the biological control of phytopathogens included 235 documents published in 151 sources (Figure 2). Scientific production grew at an annual rate of 5.7%, highlighting *Trichoderma* as a key tool in agricultural biotechnology, particularly in biological control and soil health improvement. Over the 25 years, research diversified into specialized areas such as soil microbiota modulation and omics technologies in agricultural protection. A total of 1,077 authors contributed to these papers, with an average of 5.54 co-authors per paper. However, only 29.36% of the publications included international co-authorship, indicating an opportunity to improve international collaboration, especially in regions with high agricultural potential such as Latin America, Africa, and Southeast Asia.

The documents analyzed included 15,305 references, with an average age of 6.15 years. The average number of citations per paper was 43.05, showing an uneven distribution of visibility among scientific articles; this pattern highlights the need to improve the scientific dissemination of key findings to increase the adoption of *Trichoderma* in agriculture. A total of 692 keywords were identified, reflecting the multidisciplinary nature of scientific production. Emerging areas, such as improved resistance to abiotic factors and interactions with beneficial microorganisms, remain underexplored and may represent new opportunities for future research to increase international collaboration and improve dissemination of findings to broaden the impact of *Trichoderma* in sustainable agriculture.

### 3.2 Temporal trends in scientific production and distribution of types of documents

**Figure 3:**
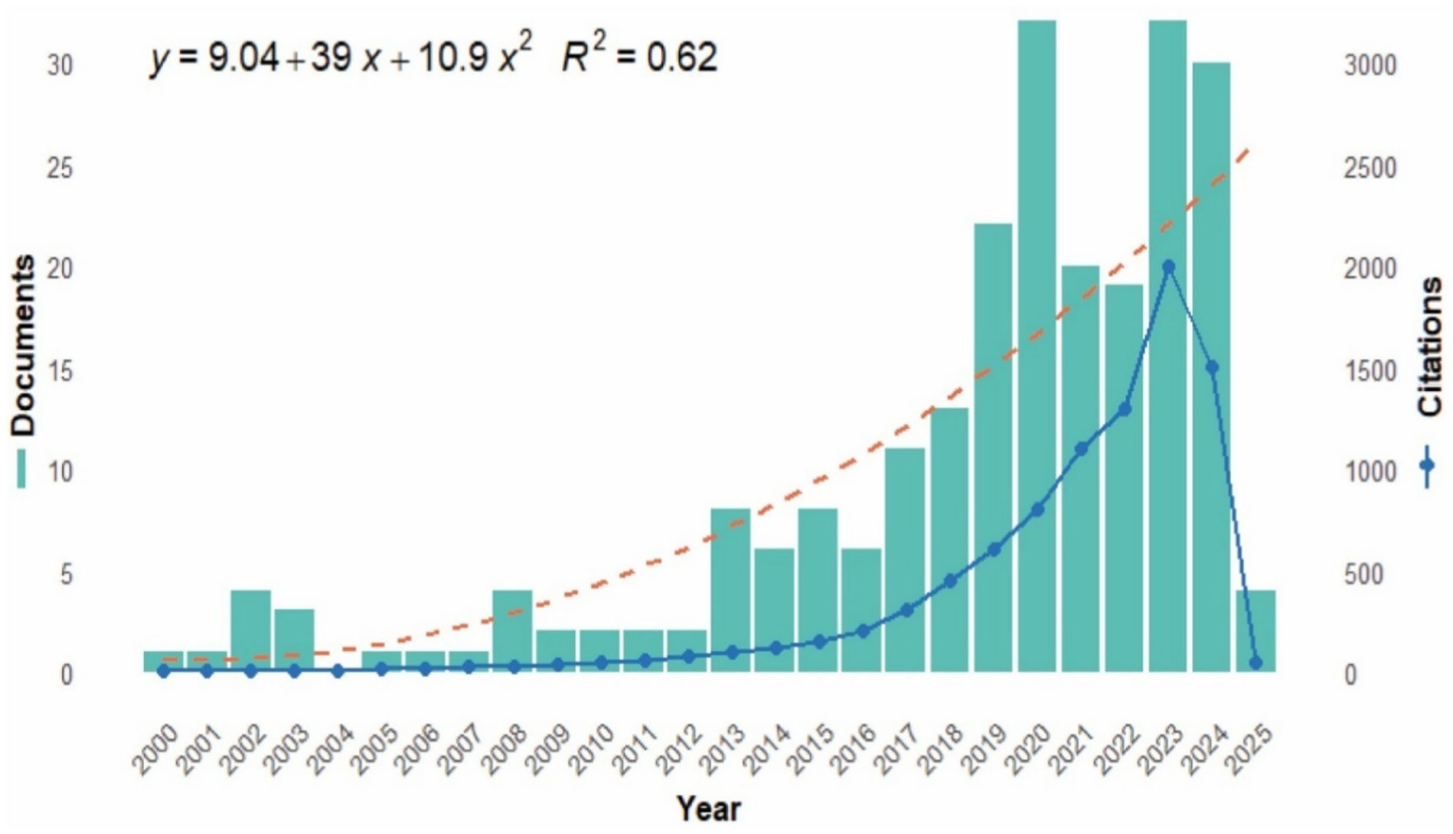
Trends in scientific production as a function of the number of publications and citations. Source: Own elaboration based on data from SCOPUS using R software.

Scientific production has increased steadily since 2000, reaching a peak of 32 publications in 2023 (Figure 3). This growth follows a second-degree polynomial model (y = 9.04 + 3.9x + 10.9x^2^; R^2^ = 0.62), which describes the acceleration of article production between 2010 and 2020, followed by a stabilization in recent years. Despite the continuous increase in the number of publications, the stabilization observed from 2020 onwards indicates a slowdown in the pace of scientific production in the most researched fields. The cumulative citations also show a similar evolution, reaching almost 3,000 citations in 2023, with fluctuations in 2024 and 2025 due to delays in indexing and the incomplete nature of the scientific production in 2025.

Of the 235 publications analyzed, 75% (180) were original articles, followed by systematic reviews (15%, *n* = 36), book chapters (6%, *n* = 16), and editorials (0.8%, *n* = 2) (Table 1). Systematic reviews stood out with an average of 3,245 citations, indicating a high impact within the thematic areas of research, while original articles accumulated 6,765 citations, with an h-index of 41. These data confirm the central role of original articles in generating knowledge in the *Trichoderma* field, with a more immediate impact in terms of citations. In contrast, book chapters and editorials had a significantly lower impact, with 93 and 2 citations, respectively. This low number of citations is related to the limitations of these types of scientific papers, which do not contribute significantly to the dissemination of experimental knowledge or to the formulation of new hypotheses.

**Table 1:** Distribution of document types, total citations, and h-index of publications.

Type of document	Publications	Percentage (%)	Total citations^*^	h index
Original article	180	75	6,765	41
Systematic review	36	15	3,245	21
Book chapter	16	6	93	6
Editorial	2	0.8	2	1
Conference paper	1	0.4	9	1
Short survey	1	0.4	63	1

* Source: Own elaboration based on data from SCOPUS.

The distribution of paper types reflects the predominance of original articles in the scientific production on *Trichoderma*. This trend is also consistent with the general pattern in experimental research areas. Although systematic reviews have an important impact on the organization and synthesis of information, their lower number of citations compared to original articles points to the difference in the speed of adoption of experimental results compared to more consolidating approaches. Finally, although the increase in scientific production and in the number of citations in the last decade indicates a greater interest in the subject, it is important to analyze and understand whether this growth is associated with an increased awareness of the benefits and challenges of biological control by *Trichoderma* and its contribution to agricultural sustainability.

### 3.3 Scientific collaboration networks and geographic distribution

**Figure 4:**
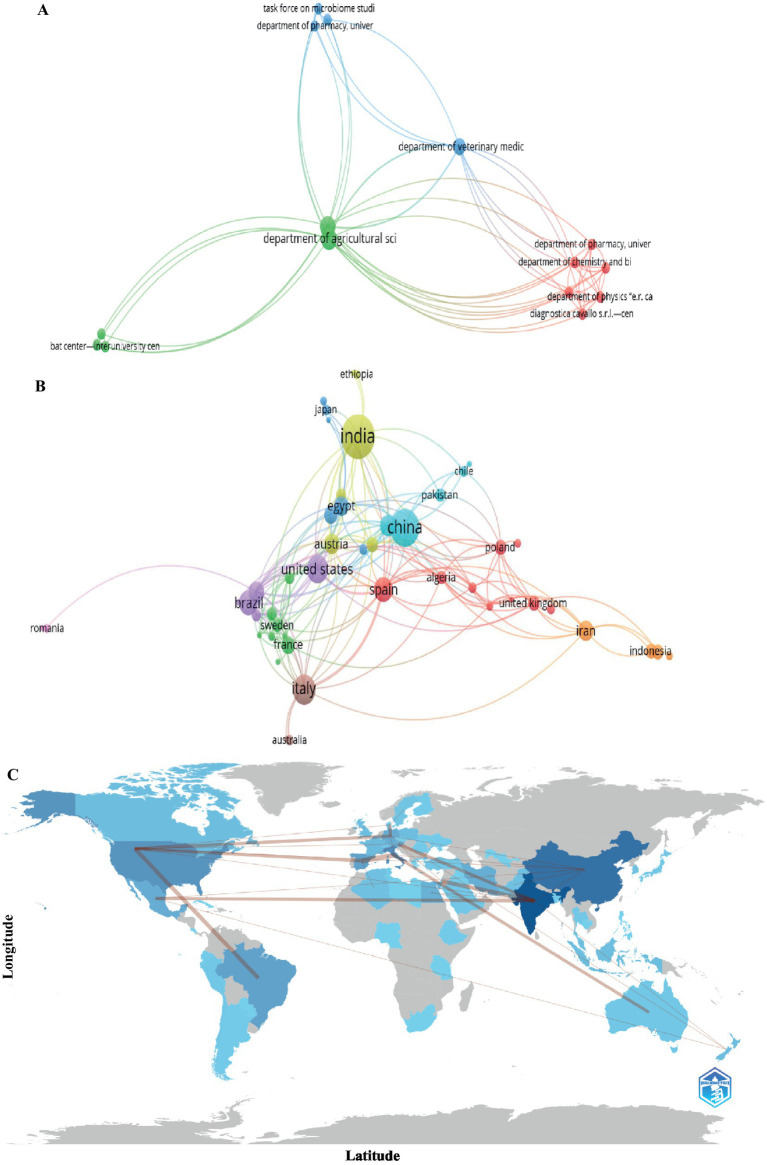
Scientific collaboration and global distribution of research on *Trichoderma* and its secondary metabolites. **(A)** Institutional collaboration networks. **(B)** International networks of scientific collaboration. **(C)** Geographical distribution of scientific production. Source: Own elaboration based on data from SCOPUS, **(A,B)** using VOSviewer software, the **(C)** using bibliometrix softw.

The network analysis at the institutional level (Figure 4A) identified three main clusters, based on the density of connections and the size of the nodes. The cluster led by the Department of Agricultural Sciences at the University of Naples Federico II is characterized by a large central node with high scientific productivity in various research areas related to *Trichoderma* as a biological control agent. The second cluster, led by the Department of Veterinary Medicine and Animal Production at the Università degli Studi di Napoli Federico II, represents a medium-sized node, with dense connections associated with active collaboration in specialized areas within biocontrol and animal health. The third cluster, led by the Department of Pharmacy and Biology at the University of Salerno, features smaller nodes but with strong interactions, indicating a highly connected network in more specific research areas in biochemistry, genetics, and microbiology.

At the country level (Figure 4B), geographic analysis revealed three dominant clusters. India leads the first yellow cluster, with a large central node reflecting the largest amount of scientific output, and strong connections to countries such as Austria and Romania; this shows India’s growing involvement in *Trichoderma* research in Asia and its close connection to Europe. The purple cluster is led by Brazil, with medium-sized nodes and strong connections to the United States and Romania, highlighting collaboration in tropical agricultural systems. The third cluster, led by China, shows large nodes and extensive connections with Pakistan and Chile, highlighting its central role in scientific collaborations on a global scale.

Regarding the geographical distribution of scientific production (Figure 4C), India led with 50 publications, followed by China (38), Italy (24), and the United States (21). Brazil and Spain contributed 18 publications each, while Germany, Mexico, Austria, Iran, and Egypt recorded 12, 12, 11, 11, 11, 11, 11, and 10 publications, respectively. The lines connecting the countries in the figure represent the main international collaborative networks, with the strongest links between India and Austria, Brazil and the United States, and China and Pakistan. Finally, it is important to emphasize that the geographic distribution of publications and the connections between countries highlight the collective advancement of knowledge in *Trichoderma* research and its contribution to agricultural sustainability worldwide.

### 3.4 Co-occurrence of keywords, temporal evolution and conceptual structure

**Figure 5:**
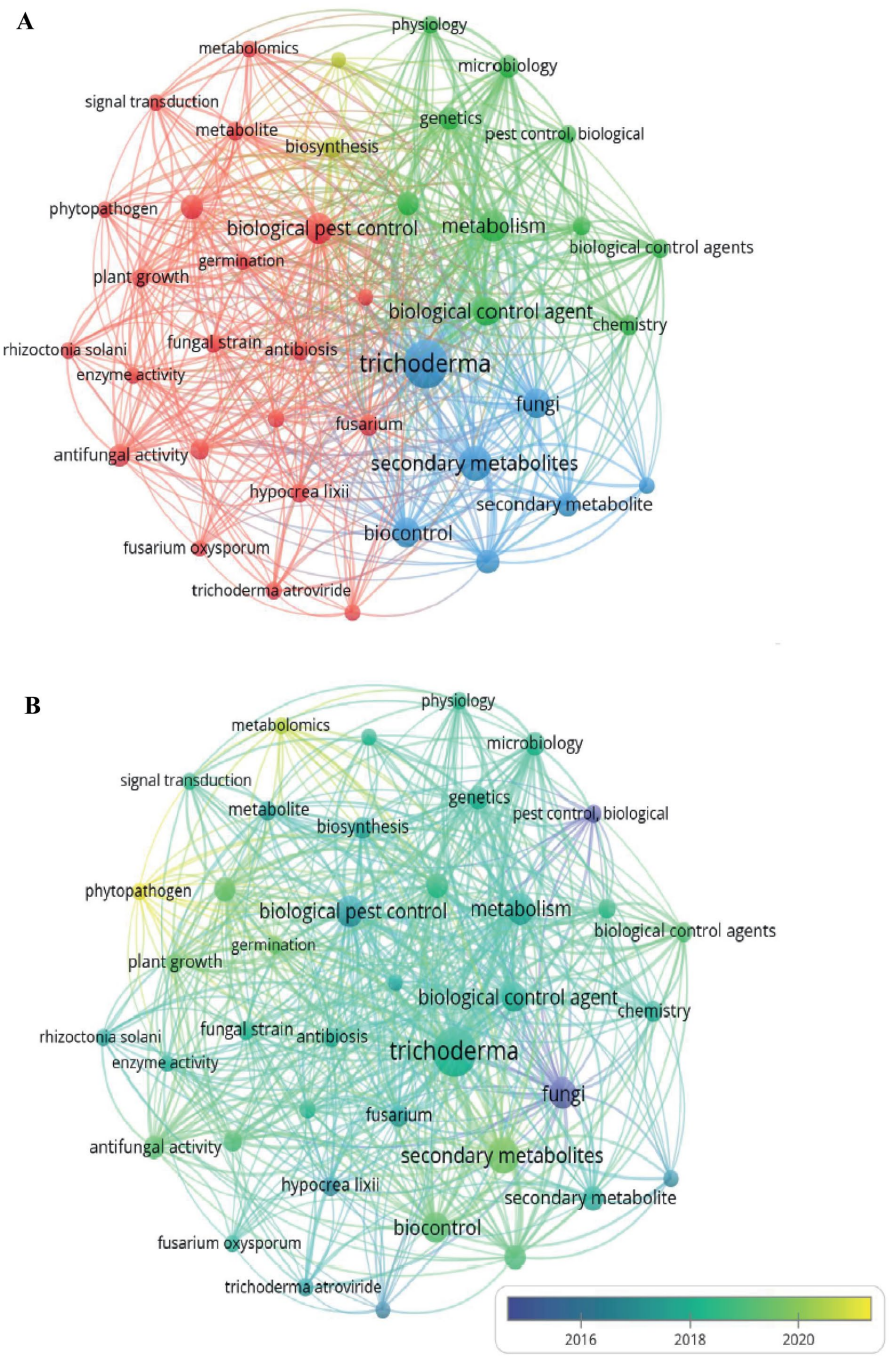
**(A)** Co-occurrence of keywords. **(B)** Temporal analysis of keywords. Source: Own elaboration based on data from SCOPUS using VOSviewer software.

The keyword co-occurrence analysis (Figure 5A) shows four thematic groups related to the biocontrol effects of secondary metabolites of *Trichoderma*. The first group, highlighted in red, includes terms related to antifungal activity and biocontrol, such as biological pest control, antifungal activity, enzymatic activity, plant growth, and phytopathogens like *Fusarium oxysporum* and *Rhizoctonia solani*. These terms emphasize the focus on *Trichoderma’s* capabilities to inhibit pathogens and promote plant growth (Mousumi Das et al., 2019; Harman et al., 2004; Diánez et al., 2016). The second group, in green, includes terms related to genetic regulation and metabolic processes, such as genetics, metabolism, and microbiology, reflecting the interest in molecular mechanisms that enable *Trichoderma* to produce secondary metabolites with biocontrol potential (Hermosa et al., 2014; Wu et al., 2018; Fang et al., 2024). The third group, in blue, focuses on terms such as secondary metabolites, biocontrol, and *Trichoderma*, highlighting the importance of these compounds in suppressing phytopathogens and enhancing plant growth (Khan et al., 2020; Reino et al., 2008).

Finally, the yellow group includes terms such as biosynthesis and phylogeny, associated with research aimed at understanding the biosynthetic pathways and phylogenetic evolution of *Trichoderma* for the identification of strains with higher efficiency in secondary metabolite production (Gutiérrez et al., 2021; Guilger-Casagrande et al., 2019; Leiva et al., 2022). his cluster distribution presents how *Trichoderma* research is integrating molecular, metabolic, and ecological approaches to optimize its use in biocontrol and enhance plant protection, with clearly defined research lines.

The temporal evolution analysis (Figure 5B) revealed a transition in *Trichoderma* research over time. In the early periods (prior to 2016), studies focused predominantly on antifungal activity and biocontrol, emphasizing terms such as fungi and biological pest control. Between 2018 and 2020, there was an increase in the frequency of terms related to molecular and biochemical approaches, such as signal transduction, genetics, and plant growth. This temporal evolution demonstrates a progressive diversification of research lines, marking a shift from studies centered on the functional interactions of *Trichoderma* to those oriented toward the characterization of its metabolic and genetic mechanisms.

**Figure 6:**
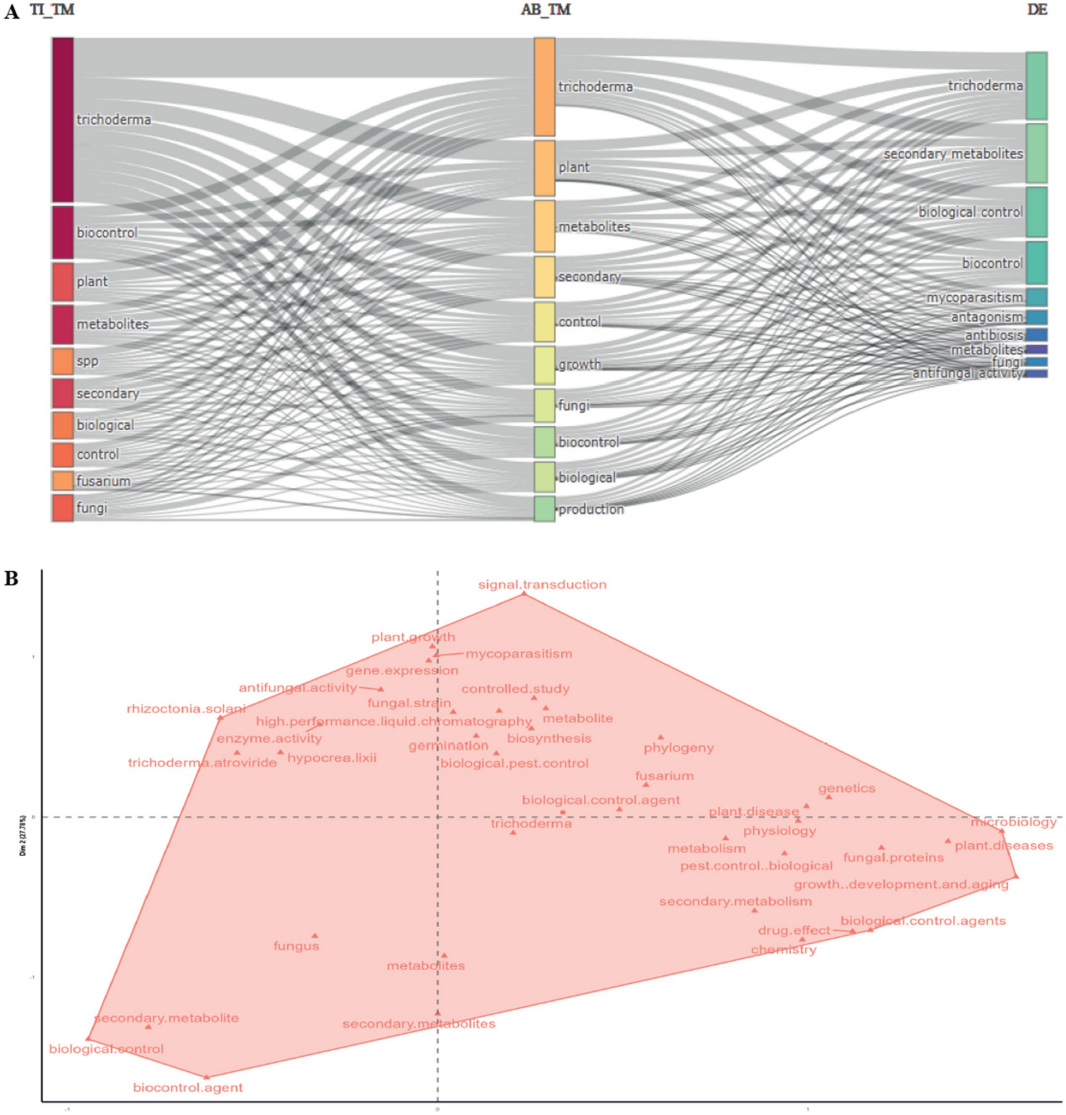
**(A)** Three-plot analysis **(B)** 5: Hierarchical clustering factor analysis. Source: Own elaborate on based on data from SCOPUS using Bibliometrix software.

The thematic trend analysis (Figure 6A) organized research topics into three main plots. The first plot grouped terms related to antifungal activity and biocontrol, which showed high frequency in the initial years of the study period. The second plot focused on the production and regulation of secondary metabolites, with a notable increase in relevance from 2018 onwards. The third plot included terms associated with molecular biology and genetics, highlighting their growing importance in recent literature. This analysis confirms the transition from applied approaches to more fundamental research focused on the molecular and biochemical mechanisms that regulate *Trichoderma*’s biocontrol activity.

The conceptual structure map obtained by factor analysis (Figure 6B) organized key terms related to *Trichoderma* along two main dimensions. Dimension 1, explaining 47.16% of the variance, grouped concepts related to biocontrol and biotechnological applications of *Trichoderma*. Dimension 2, accounting for 27.78% of the variance, focused on molecular biology and biochemical processes. Terms such as signal transduction, biosynthesis, and gene expression, located in the upper right quadrant, reflect the increasing focus on the molecular mechanisms underlying *Trichoderma*’s biocontrol activity. The proximity of secondary metabolites and biocontrol agent indicates the relevance of secondary metabolites in biotechnological applications. Experimental terms such as high-performance liquid chromatography and enzyme activity highlight the use of advanced techniques to study and optimize the metabolic processes of *Trichoderma* in agricultural biocontrol.

### 3.5 Citation network analysis: global impact, authors, and reference journals

**Figure 7:**
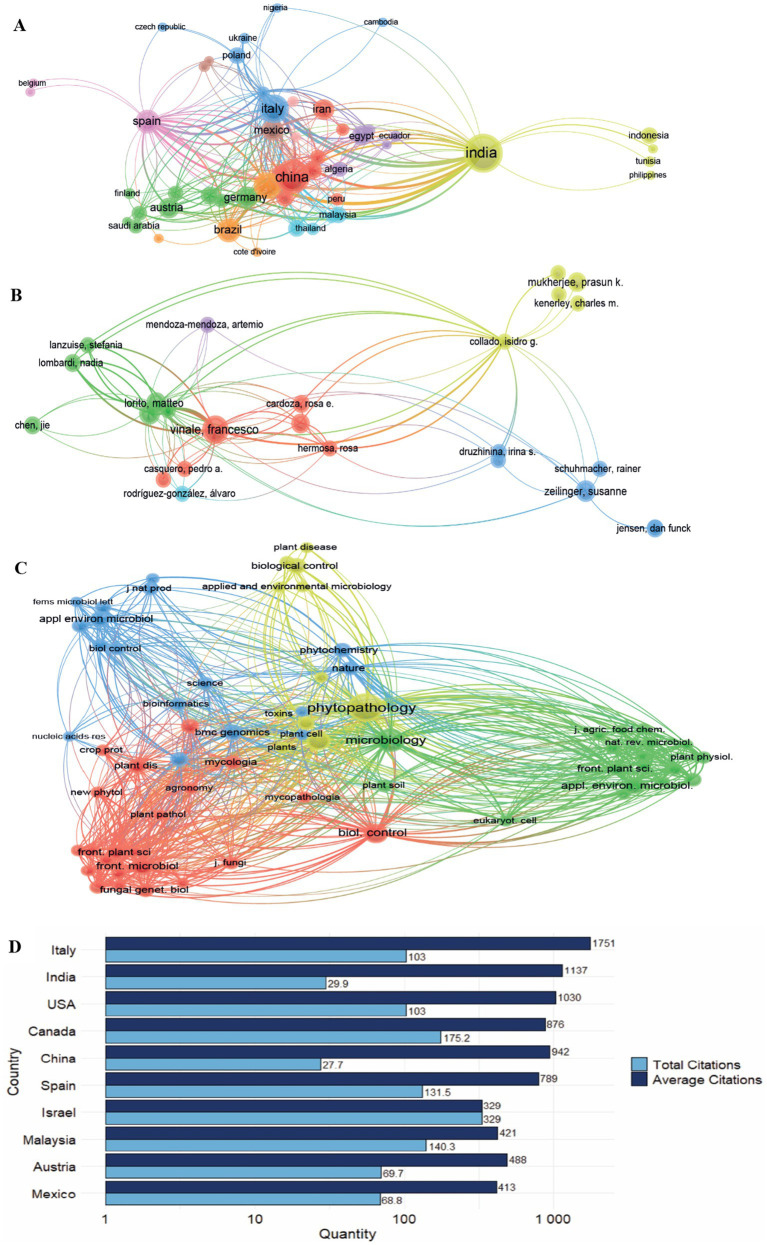
Co-citation analysis of **(A)** countries, **(B)** authors, **(C)** journals and **(D)** Ranking of countries with the highest production and their average citations. Source. Own elaboration based on data from SCOPUS using VOSviewer and R software.

The citation network (Figure 7A) is organized into clusters differentiated by color, where the size of the nodes indicates the volume of citations and the scientific influence of each country. India and China, with large nodes in the yellow and red clusters, lead scientific production, maintaining strong connections with Asian countries (Malaysia, Thailand, Indonesia) and European countries (Czech Republic, Poland). In the blue cluster, Italy and Poland, with medium-large nodes, show extensive transatlantic collaborations in agricultural and biotechnology research. The green cluster (Saudi Arabia, Germany) and the red cluster (China, Iran) contain smaller nodes, reflecting regional collaborations and emerging citation patterns. The purple cluster, which includes Egypt and Spain, represents collaborative links between Africa and Europe. This network configuration presents Asia and Europe as the primary regions for *Trichoderma* research, with collaborative links extending to Africa and Latin America.

Figure 7B shows the citation networks by author. Francesco Vinale has the highest number of citations and is positioned at the center of a large red cluster. Vinale collaborates with Matteo Lorito and Rosa E. Cardoza, forming a core group of shared references focused on the molecular and biochemical characterization of *Trichoderma* secondary metabolites and their application in biocontrol. The green cluster contains researchers such as Stefania Lanzuise and Nadia Lombardi, whose work on *Trichoderma*-phytopathogen interactions has generated substantial citations. The blue cluster, more compact and specialized, is led by Irina S. Druzhinina and Susanne Zeilinger, and focuses on genomics and metabolic regulation. The yellow cluster, more dispersed but with key links, includes Prasun K. Mukherjee and Charles M. Kenerley, whose contributions are referenced in a range of research areas, including molecular biology and agricultural applications.

Figure 7C presents the analysis of citations among journals, organized into four clusters representing areas of specialization and citation patterns in microbiology, phytopathology, and biological control. The green cluster, the largest and most interconnected, contains high-impact journals such as Applied and Environmental Microbiology and Nature Reviews Microbiology, representing studies on plant-microorganism interactions and applied biotechnology. The red cluster, more concentrated, includes journals such as Frontiers in Microbiology and Fungal Genetics and Biology, which focus on mycology and phytopathology. The blue cluster, more dispersed, connects journals in bioprospecting and microbial chemistry, such as the Journal of Natural Products and FEMS Microbiology Letters, which publish studies on the identification and characterization of secondary metabolites with biocontrol potential. The yellow cluster includes key journals such as Plant Disease and Biological Control, linking plant pathology with sustainable management practices.

Figure 7D shows the distribution of total and average citations by country. Italy leads with 1,751 total citations and an average of 103 citations per publication, followed by India (1,137 total citations) and the United States (1,030 total citations), both with an average of 103 citations per publication. Canada records 876 total citations, with an average of 175.2 citations per publication. China, with 942 total citations, has a lower average of 27.7 citations per publication. Spain has 789 total citations, with an average of 131.5 citations per publication. Countries such as Israel (329 total citations), Malaysia (421), Austria (488), and Mexico (413) have lower total citation counts, with averages ranging from 68.8 to 140.3 citations per publication. This distribution shows variation in the visibility and citation impact of research, with Canada and Malaysia having high impact per article, and China and India having higher publication volumes but lower citation averages.

The analysis of the most cited papers on *Trichoderma* (Table 2) shows that the study by Vinale et al. (2008) has 925 citations, followed by Verma et al. (2007) with 523 citations. The distribution includes original research articles (*n* = 5) and systematic reviews (*n* = 5). Experimental studies by Yedidia et al. (2003) and Howell et al. (2000) report data on the induction of systemic resistance in plants and the production of bioactive metabolites with antifungal activity. Reviews by Reino et al. (2008) and Mukherjee et al. (2013) compile information on the characterization of secondary metabolites and the genomic analysis of *Trichoderma*.

**Table 2:** Ranking of most cited papers.

Range	Author	Title	Journals	Citation	Document
1	Vinale et al. (2008)	*Trichoderma*-plant-pathogen interactions	Soil Biology and Biochemistry	925	Article
2	Verma et al. (2007)	Antagonistic fungi, *Trichoderma* spp.: Panoply of biological control	Biochemical Engineering Journal	523	Review
3	Reino et al. (2008)	Secondary metabolites from species of the biocontrol agent *Trichoderma*	Phytochemistry Reviews	459	Review
4	Zin and Badaluddin (2020)	Biological functions of *Trichoderma* spp. for agriculture applications	Annals of Agricultural Sciences	335	Review
5	Yedidia et al. (2003)	Concomitant Induction of Systemic Resistance to *Pseudomonas syringae* pv. lachrymans in Cucumber by *Trichoderma* asperellum (T-203) and Accumulation of Phytoalexins	Applied and Environmental Microbiology	329	Article
6	Mukherjee et al. (2013)	*Trichoderma* research in the genome era	Annual Review of Phytopathology	327	Article
7	Poveda et al. (2020)	Biological Control of Plant-Parasitic Nematodes by Filamentous Fungi Inducers of Resistance: *Trichoderma*, Mycorrhizal and Endophytic Fungi	Frontiers in Microbiology	282	Review
8	Howell et al. (2000)	Induction of terpenoid synthesis in cotton roots and control of Rhizoctonia solani by seed treatment with *Trichoderma* virens	Phytopathology	261	Article
9	Keswani et al. (2014)	Unraveling the efficient applications of secondary metabolites of various *Trichoderma* spp.	Applied Microbiology and Biotechnology	242	Review
10	Vinale et al. (2006)	Major secondary metabolites produced by two commercial *Trichoderma* strains active against different phytopathogens	Letters in Applied Microbiology	238	Article

*Source: Own elaboration based on data from SCOPUS.

Publications are concentrated in high-impact journals such as Soil Biology and Biochemistry, Annual Review of Phytopathology, and Applied and Environmental Microbiology. The temporal range of publications spans from 2000 to 2024, with an increase in citations in recent years. Recent studies, including those by Zin and Badaluddin (2020) on the biological functions of *Trichoderma* in agriculture, and Poveda et al. (2020) on its role in the biological control of plant-parasitic nematodes through the induction of resistance, contribute to the body of knowledge on *Trichoderma*’s use in improving plant health and agricultural productivity.

### 3.6 Scientific production of authors and their influence

**Figure 8:**
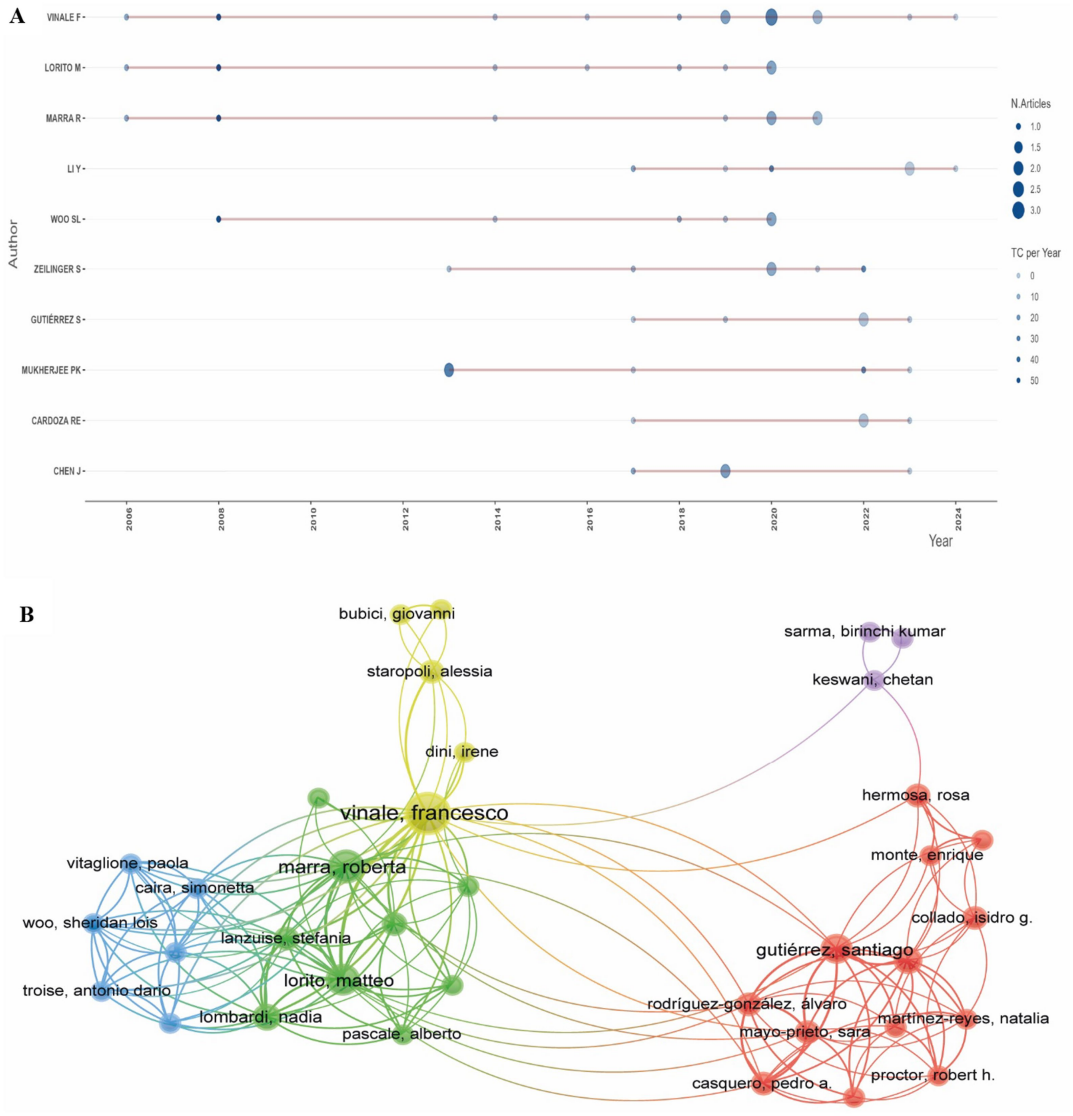
**(A)** Ranking of most productive authors and **(B)** Network analysis of the main co-authors. Source: Own elaboration based on data from SCOPUS using VOSviewer software.

Scientific production on *Trichoderma* secondary metabolites in biocontrol has experienced a continuous growth since 2006, with a significant concentration of publications in recent years (Figure 8A). Francesco Vinale leads the scientific production with 14 publications, followed by Matteo Lorito and Roberta Marra, each with 8 publications. Other authors, such as Sheridan L. Woo, Prasun K. Mukherjee and Santiago Gutierrez, have increased their production in recent years, showing a trend of research on gene regulation and biosynthesis of secondary metabolites. Although Susanne Zeilinger and Dan F. Jensen have a smaller number of publications, their contributions have been fundamental in the study of the interaction between *Trichoderma* and the soil microbiota.

In the co-authorship network (Figure 8B), five main clusters are identified, each characterized by different connection densities. The yellow cluster, with the highest density, groups Francesco Vinale, Bubici Giovanni, and Starapoli Alessia, forming a central collaboration network focused on biological control and interactions with beneficial microorganisms in agriculture (Vinale et al., 2008; Prigigallo et al., 2023). The blue cluster, which includes Vitaglione Paola and Sheridan L. Woo, maintains a high connection density within the cluster but shows limited collaboration with other groups. This cluster is associated with research on plant biostimulation, performance parameters, and the eco-physiological processes of *Trichoderma* in sustainable agriculture (Woo et al., 2023; Woo et al., 2014).

The green cluster, composed of Roberta Marra and Matteo Lorito, focuses on research related to the functionality of bioactive metabolites in plant physiology (Marra et al., 2019); as well as the characterization of the transcriptome and metabolome of *Trichoderma* in its biotic interactions within the ecosystem (Lorito et al., 2010). The red cluster, which includes researchers such as Santiago Gutiérrez and Rosa Hermosa, concentrates on the study of *Trichoderma* genes involved in plant interactions, particularly those that promote growth and enhance resistance to both biotic and abiotic stress. These studies have analyzed the expression of genes such as ThPG1 and hsp70, which contribute to improving plant defense capacity against pathogens and adverse conditions, including heat and osmotic stress. Research by Morán-Diez et al. (2009), Montero-Barrientos et al. (2010), and Hermosa et al. (2012) has reported that the manipulation of these genes in plants can increase resistance levels, contributing to the development of crops with improved tolerance to diseases and extreme environmental conditions.

The purple cluster, which includes Sarma Kumar and other authors, contains smaller yet complementary contributions. The majority of influential authors are affiliated with institutions in Italy and Spain. In recent years, there has been an increase in scientific output from researchers in India, with contributions to the field of biological control (Manzar et al., 2022; Manzar et al., 2024; Kashyap and Manzar, 2025; Manzar et al., 2021).

Citation indicators (Table 3) indicate that Francesco Vinale, Matteo Lorito, and Roberta Marra hold the highest citation counts, with 1,755, 1,533, and 1,408 citations, respectively. Although Sheridan L. Woo and Prasun K. Mukherjee have fewer publications, their citation-to-publication ratio is comparatively high. For example, Woo has 1,246 citations across 6 publications, and Mukherjee has 537 citations from 5 publications. These data provide an overview of author productivity and the citation impact of their contributions within the field.

**Table 3:** Metrics of most productive authors.

Author	Documents	h index	g index	m index	TC	PY home
Vinale Francisco	14	12	14	0,6	1,755	2006
Lorito Matteo	8	8	8	0,4	1,533	2006
Marra Roberta	8	8	8	0,4	1,408	2006
Woo Sheridan L.	6	6	6	0,333	1,246	2008
Zeilinger Susana	6	6	6	0,462	356	2013
Gutiérrez Santiago	5	4	5	0,444	67	2017
Mukherjee P. Kumar	5	4	5	0,308	537	2013
Jensen Dan F.	4	4	4	0,308	282	2013
Cardoza Rosa Elena	4	3	4	0,333	31	2017
Horwitz Benjamin A.	4	4	4	0,231	466	2013

Documents: Total publications; h index: Articles with ≥ h citations; g index: Articles with ≥ g^2^ citations; m index: h index/years of activity; TC: Total citations; PY home: Year of first publication. *Source: Own elaboration based on data from SCOPUS.

### 3.7 Bibliometric journal analysis

**Figure 9:**
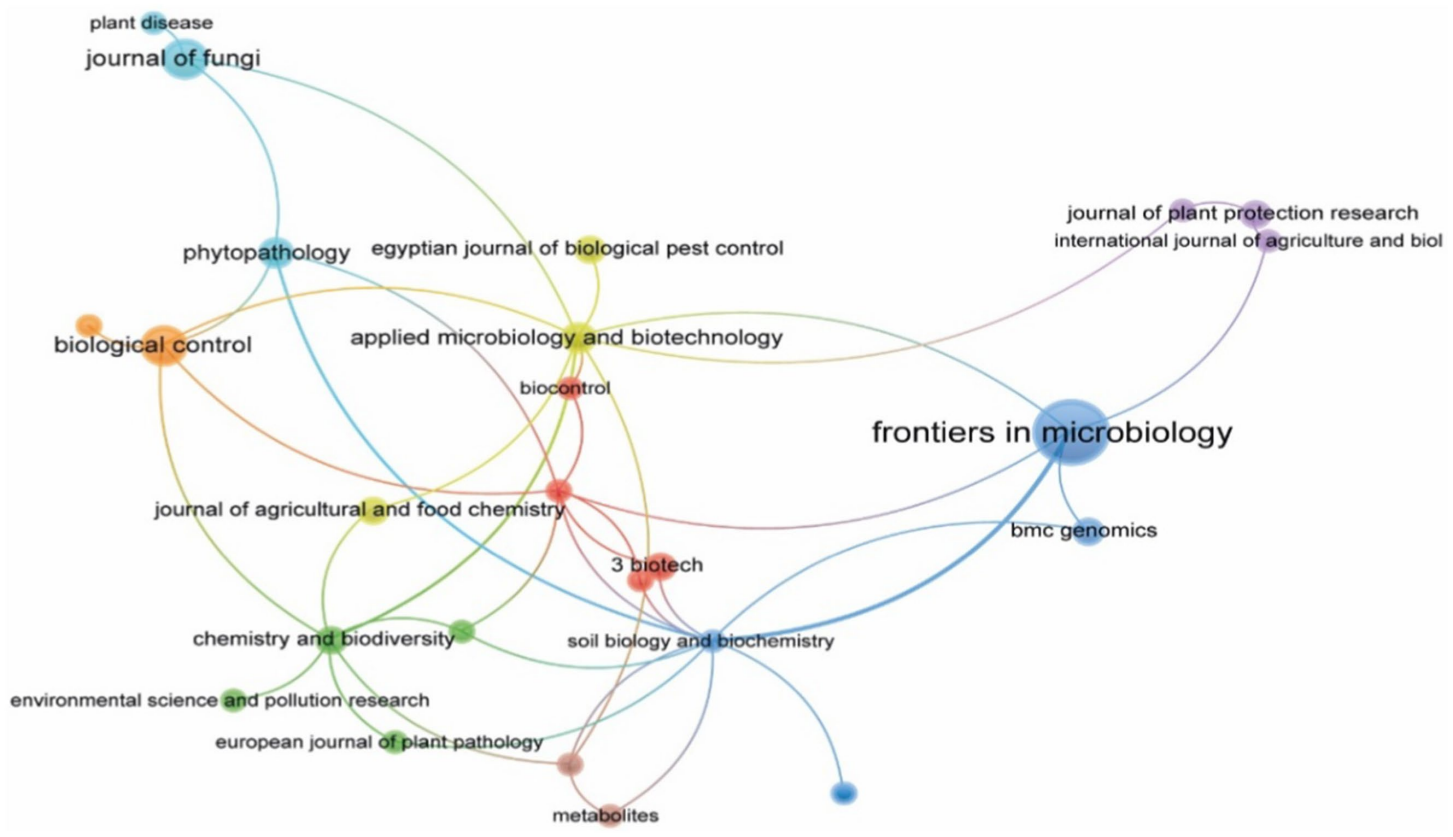
Network analysis of main scientific journals. Source: Own elaboration based on data from SCOPUS using VOSviewer software.

The bibliometric network of journal co-citation (Figure 9) presents a structure in which Frontiers in Microbiology is the most central node, showing strong interconnections with journals focused on microbiology and biotechnology, including BMC Genomics and Applied Microbiology and Biotechnology. Thematic clusters are identified: the blue cluster includes journals dedicated to general microbiology and genomics; the red cluster is oriented towards applied biotechnology and soil biochemistry; the green cluster focuses on soil chemistry, biodiversity, and environmental contaminants; and the orange cluster relates to biological control and phytopathology. The connections among these clusters reflect an interdisciplinary structure in biocontrol research, with biotechnology and microbiology linked to the understanding and application of *Trichoderma*-based strategies.

The bibliometric metrics of the ranking of journals with the highest scientific production (Table 4) indicate that Frontiers in Microbiology has the highest number of documents (21), h-index (14), and total citations (1,030). Biological Control and Journal of Fungi have an h-index of 4 and total citations of 85 and 92, respectively. The m-index analysis shows that Frontiers in Microbiology has a citation rate of 1.4. Journals such as 3 Biotech and Phytopathology have a lower publication volume but a relatively high number of accumulated citations, reflecting their specific contribution to the development of biocontrol strategies. Scientific production in the field of *Trichoderma*-based biological control is concentrated in medium-to high-impact journals, including applied studies and fundamental research in microbiology and phytopathology.

**Table 4:** Ranking metrics of the journals with the highest scientific production.

Source	Documents	h index	g index	m index	TC	PY start
Frontiers in microbiology	21	14	21	1,4	1,030	2016
Biological control	7	4	7	0,667	71	2020
Journal of fungi	7	3	7	0,6	85	2021
Applied microbiology and biotechnology	4	4	4	0,333	353	2014
International journal of molecular sciences	4	3	4	0,375	92	2018
Phytopathology	4	4	4	0,154	469	2000
3 biotech	3	3	3	0,375	55	2018
Agronomia mesoamericana	4	1	2	0.143	7	2019
BMC genomics	3	3	3	0,231	334	2013
Chemistry and biodiversity	3	3	3	0,167	172	2008

Documents: Total publications; h index: Articles with ≥ h citations; g index: Articles with ≥ g^2^ citations; m index: h index/years of activity; TC: Total citations; PY home: Year of first publication. *Source: Own elaboration based on data from SCOPUS.

### 3.8 Theme in trends

**Figure 10:**
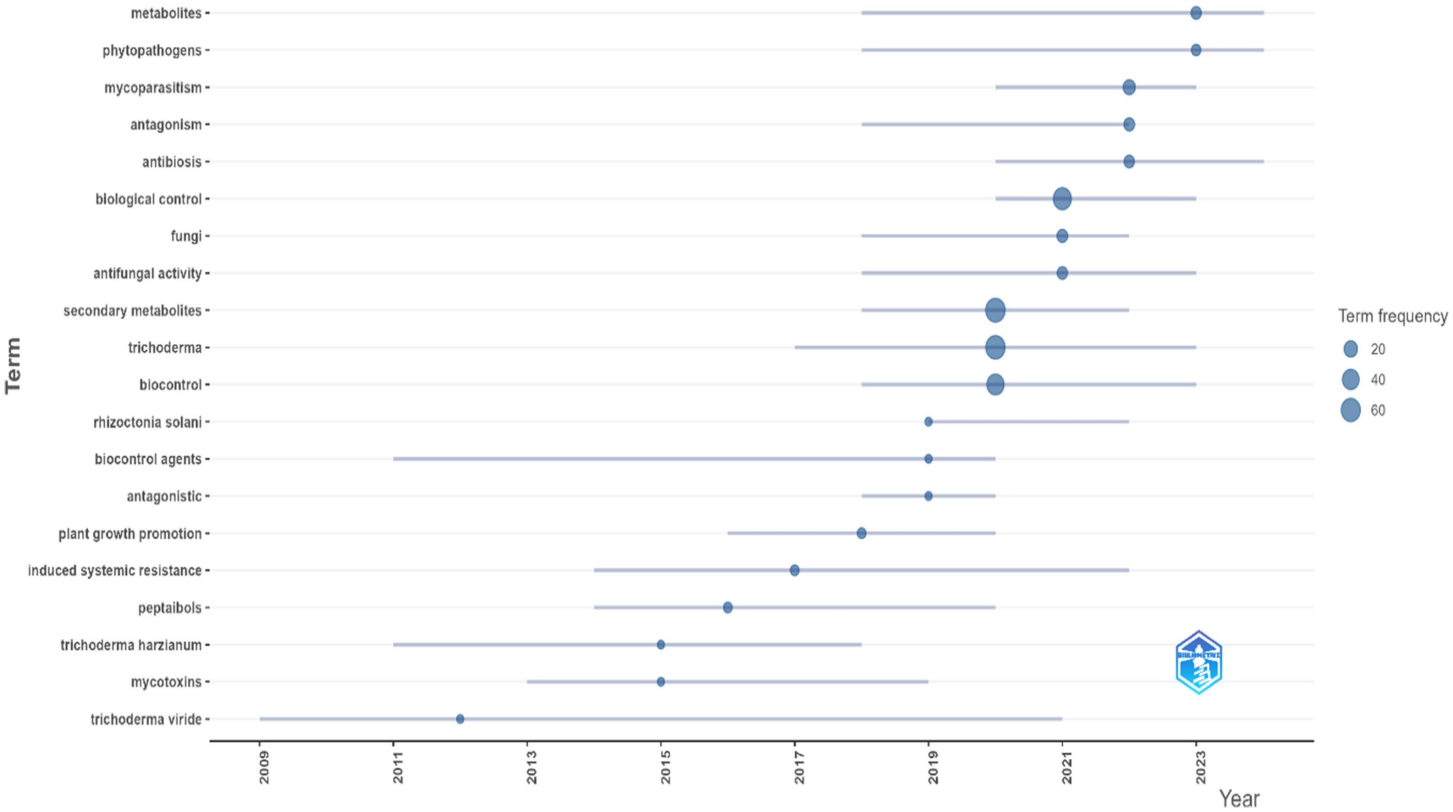
Keyword evolution analysis. Source: Own elaboration based on data from SCOPUS using VOSviewer software.

Figure 10 presents the temporal dynamics of key research terms in the study topic. Since 2000, terms such as metabolites, plant pathogens, and antagonism have increased in frequency, corresponding to an initial focus on direct biocontrol mechanisms, including the production of antifungal compounds and ecological competence (Vinale et al., 2008; Verma et al., 2007). From 2016 onwards, there is an increase in the frequency of terms such as antifungal activity, induced systemic resistance (ISR), and bioactive peptides, associated with a transition toward molecular studies and plant–microorganism interactions (Shoresh et al., 2010; Poveda et al., 2020).

Species such as *T. harzianum* and *T. viride* maintain a consistent presence in the literature and the biofungicide market, linked to their efficacy in controlling pathogens such as *Fusarium oxysporum* and *Rhizoctonia solani* (Mousumi Das et al., 2019; Manzar et al., 2021). This trend corresponds with the increasing demand for sustainable alternatives to the excessive use of agrochemicals, which has led to efforts to reduce microbial resistance and the environmental impact associated with synthetic pesticide residues (María Suaste, 2020; Ribeiro et al., 2001).

### 3.9 Efficacy of *Trichoderma* spp. as a biocontrol agent

The comprehensive analysis of the 235 scientific publications on *Trichoderma* as a biocontrol agent indicates a complex interaction of biological, environmental, and technological factors. Data indicate that *T. asperellum* achieves an efficacy of 92.2% against Colletotrichum gloeosporioides in tomato crops, primarily through the production of antifungal metabolites (peptaibols, 2-aminoisobutyric acid) and rapid colonization of the rhizosphere (Alfaro-Vargas et al., 2022). In contrast, efficacy is reduced to 53% against oomycetes such as *Phytophthora infestans* on potato, due to differences in cell wall composition (Alfiky et al., 2024). Biocontrol efficacy varies between controlled and field conditions: *in vitro* studies report control levels of 80–100%, whereas field studies show lower efficacy, typically ranging from 40–60%. Reductions in efficacy are more pronounced in clay soils (35% lower than in loam soils) and under elevated temperatures (>30°C) (Shao et al., 2025; Zin and Badaluddin, 2020).

The variation in biocontrol efficacy is influenced by environmental factors such as soil pH, soil texture, and the presence of competing microorganisms variables that are rarely considered in current experimental designs (Poveda et al., 2020). Additionally, methodological limitations are identified: only 18% of the studies evaluate commercial formulations or optimized application strategies, and few investigate advanced methods such as nanoencapsulation of metabolites (Shao et al., 2025). These aspects contribute to the gap between laboratory results and practical applications in agricultural systems.

## 4 Discussion

To date, no comprehensive bibliometric analysis specifically addresses the scientific production on *Trichoderma* secondary metabolites and their application in the biological control of plant pathogens. This gap in the literature limits the identification of emerging trends, international collaborations, and priority research areas in this field. The present study provides a mapping of the evolution of *Trichoderma* secondary metabolite research using bibliometric tools such as VOSviewer and Bibliometrix.

Among the most extensively studied metabolites are peptaibols—non-ribosomal peptides containing *α*-aminoisobutyric acid and a C-terminal alcohol. These compounds disrupt the permeability of phytopathogenic fungal cell membranes, inducing osmotic collapse that compromises viability (Alfaro-Vargas et al., 2022; Zhang et al., 2022; Zeilinger et al., 2016). Their antifungal activity has been demonstrated against pathogens of economic relevance, including *Fusarium oxysporum* and *Botrytis cinerea* (Shi et al., 2012). Terpenoids, derived from the mevalonic acid pathway, exhibit dual functionality: direct antifungal activity and modulation of rhizosphere microbial interactions that contribute to soil microbiome stability (Howell et al., 2000; Shi et al., 2020). Additionally, terpenoids stimulate phytoalexin production in plants, enhancing systemic resistance (Chou et al., 2023). Pyrones and pyrazoles display complementary antimicrobial properties and can activate plant defense mechanisms (Abd-Elhalim et al., 2025). These compounds are involved in both systemic acquired resistance (SAR) and induced systemic resistance (ISR) pathways, contributing to plant protection against biotic and abiotic stresses (Shoresh et al., 2010).

Research on *Trichoderma* metabolites has expanded substantially over the past two decades, particularly in the characterization of biosynthetic pathways and antifungal mechanisms (Savas et al., 2021; Vinale et al., 2008; Yan and Khan, 2021). However, a substantial portion of this research has been conducted under controlled laboratory conditions, with limited validation in field settings. Further studies are needed to assess biocontrol efficacy under variable environmental, strain, and species conditions (Poveda et al., 2020; Zeilinger et al., 2016; Brunner et al., 2003). This is relevant because biocontrol efficacy depends on tripartite interactions among strain, environment, and pathogen, which require evaluation in diverse agroecological systems.

An additional limitation is the limited number of studies on the metabolite-mediated modulation of the rhizosphere microbiome. While 85% of publications document direct antagonism against plant pathogens (Alfiky et al., 2024; López-Valenzuela et al., 2022; Stecca et al., 2014), fewer than 15% examine the impact on soil microbial networks (Chou et al., 2023; Mendoza-Mendoza et al., 2024). Expanding research on these interactions is essential for optimizing *Trichoderma* applications in agricultural systems, where the soil microbiota plays a critical role in plant health and biocontrol agent performance (Druzhinina et al., 2011; Kredics et al., 2018; Kredics et al., 2024). Addressing these gaps requires an integrated framework combining metabolomics, microbiome analysis, and precision agronomy.

The bibliometric analysis follows a second-degree polynomial model (R^2^ > 0.85), characterized by an accelerated growth phase (2005–2015) and subsequent stabilization (Figure 3) (Poveda et al., 2020; Zin and Badaluddin, 2020; Vinale and Sivasithamparam, 2020). Publications from 2020 to 2023 account for 45% of total citations (approximately 3,000), representing a substantial portion of current research output. Citation distribution, however, remains uneven: 70% of molecular studies achieve high visibility (h-index > 15), while only 30% of applied research reaches similar impact levels, indicating persistent challenges in technology transfer. Research on *Trichoderma* secondary metabolites is predominantly concentrated in Asia and Europe, with a relatively low number of contributions from developing countries where *Trichoderma* applications could have agronomic relevance (Figure 4C). Countries such as India and China lead in publication volume, with 50 and 38 publications respectively, while their bibliometric impact is distributed through international collaborations (Figure 4B). Italy, with 24 publications, shows a higher citation density. This distribution indicates differences in citation metrics, suggesting the need for increased North–South collaborations to support technology transfer and validation studies in diverse agroecological conditions, particularly in tropical regions where bioinputs may have the greatest impact.

The main affiliations contributing to *Trichoderma* research include institutions from Italy, Brazil, and France. The Consiglio Nazionale delle Ricerche (Italy) leads with 17 publications, followed by the Università degli Studi di Napoli Federico II (14 publications) and the Istituto per la Protezione Sostenibile delle Piante (12 publications) (Table 5). These institutions have established collaborations with countries in Latin America and Asia, facilitating the exchange of knowledge and the development of context-specific solutions. Citation impact is unevenly distributed, with publications from Europe and North America receiving more citations. This pattern may reflect differences in international visibility, while studies from Asia have a lower citation rate despite increasing research output. In Latin America, the participation of Brazil and Mexico in *Trichoderma* research indicates an expansion of agricultural biotechnology and its integration into bioprotection strategies at a global level. The observed geographical distribution supports the need to strengthen international collaborations, particularly in regions where *Trichoderma* technologies could enhance agricultural systems (Tyśkiewicz et al., 2022). Collaborative, multidisciplinary approaches can facilitate knowledge exchange, cross-validation of results, and the development of solutions tailored to the agroecological characteristics of each region (Torres-De La Cruz et al., 2015).

**Table 5:** Ranking of scientific production of institutions.

Affiliation	Documents	Country
Consiglio Nazionale delle Ricerche	17	Italy
Università degli Studi di Napoli Federico II	14	Italy
Istituto per la Protezione Sostenibile delle Piante, CNR	12	Italy
Embrapa Recursos Geneticos e Biotecnologia	7	Brazil
CNRS Centre National de la Recherche Scientifique	7	France
Shanghai Jiao Tong University	7	China
Chinese Academy of Agricultural Sciences	6	China
Universidad de Salamanca	6	Spain
Universität Innsbruck	5	Austria
Technische Universität Wien	5	Austria

*Source: Own elaboration based on data from SCOPUS.

The co-occurrence analysis of key terms (Figure 5A) identifies four main clusters: antifungal activity and biocontrol (Reino et al., 2008; Benítez et al., 2004), gene regulation and metabolic processes (Błaszczyk et al., 2016; Steyaert et al., 2003); secondary metabolite production; and biosynthesis and phylogeny (Contreras-Cornejo et al., 2016; Mona et al., 2017; Tchameni et al., 2020). The interconnections between these clusters indicate thematic integration in *Trichoderma* research, with a focus on the relationship between secondary metabolite production and pathogen suppression in agricultural systems. The temporal evolution of key terms (Figure 5B) shows a progression from studies focused on antifungal activity and biocontrol to research exploring molecular and biochemical processes, including signal transduction and genetics. This trend illustrates the diversification of research topics, transitioning from studies on functional interactions of *Trichoderma* toward characterization of metabolic and genetic mechanisms.

The citation analysis by author (Figure 7B) identifies Francesco Vinale as the most cited author, forming a central collaboration network with Matteo Lorito and Rosa E. Cardoza in *Trichoderma* research (Harman et al., 2004; Woo et al., 2014; Lorito et al., 2010; Vinale et al., 2013). Their work has contributed to understanding the regulation and application of *Trichoderma* secondary metabolites in biological control, gene regulation, and secondary metabolite biosynthesis (Sherkhane et al., 2017). Citation metrics reflect the individual impact of each author and indicate collaborative networks across disciplines and geographic regions.

Future research should prioritize the field validation of *Trichoderma* metabolites and the development of application protocols that account for region-specific environmental conditions and microbial interactions (Abbas et al., 2022; Shenouda and Cox, 2021). The stability of these metabolites, their compatibility with other bioinputs, and their efficacy in different soil types and climatic conditions have not been systematically addressed in existing studies. Addressing these aspects is necessary for effective technology transfer from research settings to agricultural systems. Interdisciplinary collaborations involving biotechnology, agronomy, and industry are essential to optimize production processes and fermentation protocols required for the effective commercialization of *Trichoderma*-based products.

Future research may also integrate approaches such as functional genomics, metabolomics, and soil microbiome analysis to develop predictive models applicable to agricultural environments (Druzhinina and Kubicek, 2017; Friedl and Druzhinina, 2012; García-Estrada et al., 2018). Technological developments, including gene editing, nanotechnology, and advanced fermentation methodologies, could support improvements in the stability and scalability of *Trichoderma* metabolites, facilitating their integration into sustainable agricultural systems. Research in this area should adopt a holistic perspective, considering both metabolite production and their role in agricultural ecosystems.

## 5 Conclusion

This bibliometric analysis outlines the evolution of research on *Trichoderma* secondary metabolites, mapping thematic areas, collaborative networks, and methodological trends over the past 25 years. The analysis presents an increased focus on molecular and genetic studies but limited progress in field-scale validation, formulation development, and integration with microbial ecology. Most research outputs originate from Asia and Europe, while contributions from regions where *Trichoderma*-based biocontrol could have considerable agronomic relevance remain underrepresented. The study provides a structured overview of existing knowledge and identifies areas requiring further research, particularly the need for experimental validation under diverse agroecological conditions and the development of scalable production and application strategies. Advancing this field will require interdisciplinary approaches that integrate genomics, metabolomics, microbiome studies, and precision agriculture technologies with applied agronomy to optimize the production, stability, and performance of *Trichoderma* metabolites in sustainable agricultural systems.

## Data Availability

The raw data supporting the conclusions of this article will be made available by the authors, without undue reservation.
